# Operative versus nonoperative treatment in children with painful rigid flatfoot and talocalcaneal coalition

**DOI:** 10.1186/s12891-020-03213-5

**Published:** 2020-03-24

**Authors:** Giovanni Luigi Di Gennaro, Stefano Stallone, Eleonora Olivotto, Paola Zarantonello, Marina Magnani, Tullia Tavernini, Stefano Stilli, Giovanni Trisolino

**Affiliations:** 1 Pediatric Orthopedics and Traumatology, IRCCS Istituto Ortopedico Rizzoli, Bologna, Italy; 2 RAMSES Laboratory, RIT Department, IRCCS Istituto Ortopedico Rizzoli, Bologna, Italy

**Keywords:** Tarsal coalition, talocalcaneal, Flatfoot, Child, Surgical treatment, Manipulation under anesthesia, Allograft, Arthroereisis

## Abstract

**Background:**

The management of painful rigid flatfoot (RFF) with talocalcaneal coalition (TCC) is controversial. We aimed to compare operative and nonoperative treatment in children with RFF and TCC.

**Methods:**

We retrospectively reviewed medical records and radiographs of children with RFF and TTC treated between 2005 and 2015. The nonoperative treatment consisted of manipulation under anesthesia, cast immobilization and shoe insert after cast removal. The operative treatment consisted of combined TCC resection, graft interposition and subtalar arthroereisis.

**Results:**

Thirty-four children (47 ft) in the nonoperative group and twenty-one children (34 ft) in the operative group were included. No differences were found between groups, concerning baseline characteristics. The mean age at treatment was 11.8 years (9–17): 11.6 (9–17) for the nonoperative group, 12.2 (10–15) for the operative group. The mean follow-up averaged 6.6 (3–12) years and was significantly longer in the nonoperative group (7.8 versus 4.7 years; *p* < 0.0005), since the operative procedure was increasingly practiced in the latest years.

There were no complications in either groups, but 6 patients (7 ft) in the nonoperative group were unsatisfied and required surgery. At the latest follow-up, the AOFAS-AHS improved in both groups, although the operative group showed significantly better improvement. The operative group reported also significantly better FADI score, after adjustment for follow-up and baseline variables.

**Conclusion:**

The operative treatment showed better results compared to the nonoperative treatment. Symptomatic RFF with TCC in children can be effectively treated in one step with resection, graft interposition and subtalar arthroereisis. Further prospective randomized studies are needed to confirm our findings and to identify the best operative strategy in this condition.

## Background

The congenital tarsal coalition is a partial or complete fusion between two or more midfoot or hindfoot bones, due to abnormal formation of bone, cartilage or fibrous tissue [1].

The incidence of tarsal coalition is about 1%, although, being often asymptomatic, the true prevalence is around 13% [2–6], with a male predominance and bilaterality in 50% of cases [1]. The talocalcaneal coalition (TCC) and the calcaneo-navicular coalition (CNC) are the most frequent compared to other types [7, 8].

Many patients with TCC typically show a rigid flatfoot (RFF) with loss of the medial arch [5, 9]. RFF must be distinguished from flexible flatfoot (FFF). FFF is a widespread idiopathic condition among children. In contrast with RFF, FFF is clinically characterised by the possibility of restoring a medial arch at physical examination when standing on tip toes or with the Jack’s test (rise of the medial arch with great toe passive dorsiflexion) [10, 11]. Compared to FFF, RFF is most frequently symptomatic [12]. Pain is present in about 25% of cases; symptoms generally start in the second decade of life, when the coalition ossifies [2, 9]. The management of symptomatic RFF with TCC is controversial [13]. Many authors agree that conservative treatment must be initially attempted, while surgery should be reserved when conservative treatment fails [1, 13–16].

Historically, subtalar or triple arthrodesis has been recommended for pain relief [17, 18]. More recently, some authors reported good results following bar resection, possibly associated with interposition of various tissues [8, 19–24]. This treatment aims to relieve pain and increase subtalar motion.

Moreover, some authors stressed the importance of the correction of the hindfoot alignment, during the management of painful RFF with TCC [19, 25–29].

The aim of this study was to compare nonoperative and operative treatment in children affected by TCC and RFF.

## Methods

After institutional review board approval, a retrospective review of medical records and radiographs was conducted in patients admitted for painful RFF with TTC between 2005 and 2015 at a single tertiary referral center for pediatric orthopedics. The study involved 55 children (35 males, 20 females; 26 bilateral cases) accounting for overall 81 ft. All the patients were treated according to the surgeon’s preference and experience thus the study was not randomized. Children with painful TCC and RFF (defined “Staheli Arch Index” > 1.28 and rearfoot eversion > 10° [30, 31]) were enrolled. Computed Tomography (CT) was performed to confirm the diagnosis. We divided our cohort in two groups: A) nonoperative, consisting of manipulation under anesthesia and cast application (34 children; 47 ft); B) operative, consisting in TCC resection, graft interposition and subtalar arthroereisis (21 children; 34 ft).

We excluded from the study: children treated for idiopathic or secondary flatfoot without tarsal coalition; tarsal coalitions other than TCC; children who underwent other operations; children with syndromic pathologies or neuromuscolar disorder; children with incomplete documentation or lost to follow-up.

An Italian validated version of the American Orthopaedic Foot and Ankle Society Ankle-Hindfoot Score (AOFAS-AHS) was completed at admission for each patient [32], .Lateral talar-first metatarsal angle and calcaneal pitch were calculated on radiographs. On CT we assessed the heel valgus, the coalition area, the subtalar joint space narrowing (JSN) and the presence of osteoarthritis (OA) of the subtalar joint [19, 33–35]. The tarsal coalition was classified according to the Rozansky’s classification [19, 33, 34].

Nonoperative treatment consisted of manipulation in supination under anesthesia; then a short-leg cast in inversion was applied for 5 weeks [18]. After cast removal, patients received custom shoe inserts to reduce overpronation and support plantar arch.

The operative treatment consisted of combined TCC resection, allograft interposition and correction of the hindfoot alignment by subtalar athroereisis with a nonresorbable screw (SPHERUS talar screw, Gruppo Bioimpianti® - Milan - Italy).

### Surgical technique

**Fig. 1:**
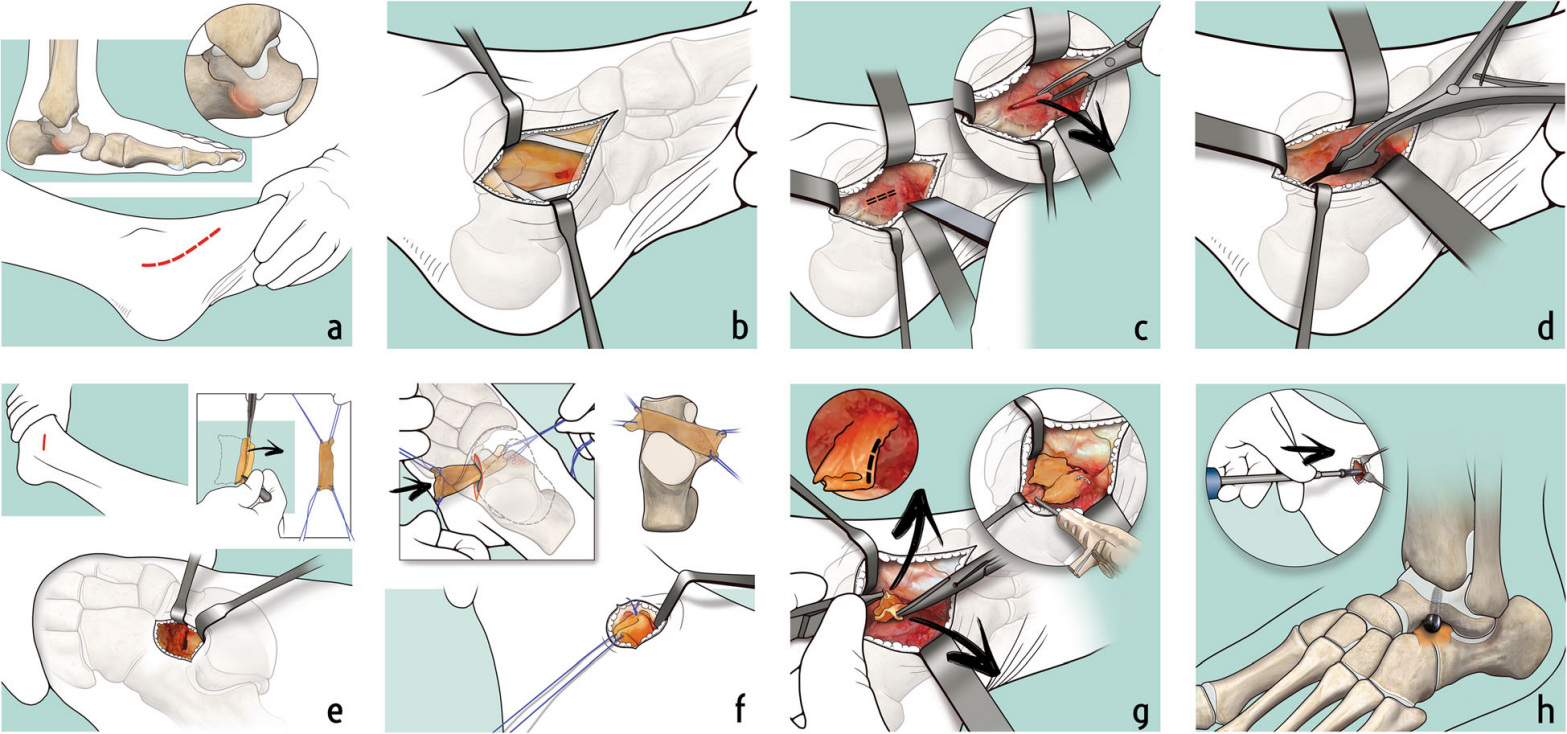
Illustrations of the surgical technique step by step. **a** A medial approach is performed with an incision over the sustentaculum tali, centered to the coalition. **b** The tibialis posterior tendon is retracted dorsally, while flexor hallucis longus and the flexor digitorum longus tendons are retracted plantarly, exposing the bone bridge. **c** After the identification of the talonavicular joint anteriorly and the residual talocalcaneal joint posteriorly, the bridge is excised using osteotomes. **d** The joint is open with a spreader, gaining the separation and complete motion of the talocalcaneal joint. **e** A lateral incision is performed over the sinus tarsi, exposing the lateral facet of the talus. A frozen fascia lata allograft is folded in two layers before positioning. **f** The fascia lata allograft is passed from lateral to medial into the tarsal canal and the two layers of the graft are placed covering the bony surfaces of the resected area. **g** The edges of the graft are fastened with suture anchors or absorbable stitches. **h** A calcaneo-stop screw is inserted in the talus to keep the correction

The patient was placed in supine position, with a pneumatic torniquet on the tight. A medial incision was performed, starting from the posterior apex of the medial malleolus, continuing for 5 cm over the sustentaculum tali, until the posterior border of the palpable navicular bone (Fig. 1a). The tendon sheath was incised longitudinally, to expose the tibialis posterior tendon, that was retracted dorsally, while flexor hallucis longus and flexor digitorum longus tendons were retracted plantarly (Fig. 1b). The deltoid ligament was dissected over the bone bridge, that was exposed, identifying the talonavicular joint anteriorly and the residual talocalcaneal joint posteriorly. The bridge was excised with an osteotome, obtaining separation and complete motion of the talocalcaneal joint (Fig. 1c). A calibrated spreader was used to obtain an adequate gap for insertion of the graft (Fig. 1d). A lateral incision was performed over the sinus tarsi, identifying the lateral facet of the talus and exposing the tarsal canal. A frozen fascia lata allograft was folded in two layers before positioning (Fig. 1e). The size of the allograft was prepared according to the size of the resected area. A blunt dissection was performed to slightly dilate the tarsal canal and facilitate the graft passage. The graft was passed into the tarsal canal and the two layers of the graft were carefully placed on the bony surfaces of talus and calcaneus at the level of the coalition, mimicking the articular surfaces of the talocalcaneal joint [36] (Fig. 1f). The edges of the graft were fastened to the surrounding bony or capsular structures, using suture anchors or absorbable stitches (Fig. 1g). Using the same lateral approach, a screw housing was prepared by a straight awl, and the body of the talus was penetrated obliquely upwards. Under fluoroscopic control, a talar screw was then inserted in the housing, until the spherical head of the screw, projecting into the sinus tarsi and resting against the floor of the latter, provided the desired degree of correction (Fig. 1h). The tension of the Achilles’ tendon was checked, and a percutaneus tendon lenghtening, was further performed, whenever the ankle did not achieve at least 5° of dorsiflexion with the knee flexed.

A plaster cast was applied for 4 weeks. After cast removal, walking with full weight bearing was allowed as soon as pain was tolerable.

The screw was removed after 2 years, if the foot increased by two or more shoe sizes.

### Follow-up

Patient were followed for at least 3 years (at least 1 year after screw removal in the operative group). Data were collected and analyzed by two independent observers. The clinical and functional outcomes were assessed by the AOFAS-AHS and the Italian validated version of the Foot and Ankle Disability Index (FADI) [37]. Both questionnaires were completed during the latest follow-up visit. Postoperative radiographs were available only in 4 cases in the nonoperative group and in 13 cases in the operative group, thus the differences between preoperative and postoperative radiographic values were not evaluated.

### Statistical analysis

Continuous data were expressed as means, whereas categorical and ordinal data were expressed as absolute values and percentages. Normality was tested using the chi-square test for categorical variables and the Kolmogorov-Smirnov test for continuous variables. Differences in baseline and outcome characteristics between groups were tested using Fisher’s exact test for categorical variables and Student’s t-test for paired and unpaired data (normal distribution) or Mann-Whitney U-test and Wilcoxon signed-rank test (skewed distribution) for continuous variables. Exploratory univariable analyses with general linear models were performed to identify potential associations among baseline variables and outcomes. Linear mixed effect models with patient as random effect were used, to avoid violation of the principle of independence in bilateral cases. Results were presented as crude and adjusted means with 95% Confidence Intervals.

Propensity analysis was used for adjustment of potential selection biases in operative decision [38]. For each patient, we estimated propensity scores (PS) for receiving nonoperative or operative treatment, using a binary logistic model that included baseline variables. The balance of the PS was checked observing the overlap in the range of propensity scores across the two treatments and comparing the quintiles. T-test showed no statistically significant differences in covariate means between groups after matching. Examining treatment effects on the outcome across PS quintiles, no association was observed between the outcome and the probability of receiving either treatment, meaning that there is no evidence of unmeasured bias. PS were used to derive inverse probability of treatment weights (IPTW), with the inverse of the propensity score for the operative group and the inverse of 1 minus the propensity score for nonoperative group. Then, the IPTW were used to adjust the difference in AOFAS-AHS and FADI between groups. Statistical significance was set at *p* < .05. All analyses were performed with SPSS v. 22.0 (SPSS, Chicago, IL, USA).

## Results

No differences were found between groups, concerning age at treatment, gender, bilaterality, baseline AOFAS-AHS, radiographic features (see Table 1).

**Table 1:** Baseline demographic, clinical and radiographic data

**Baseline variable**	**Group A (nonoperative)**	**Group B (operative)**	***p-value***
N° of children (feet)	34 (47)	21 (34)	.07
Male/female ratio	23/11	12/9	.35
Age (years) [mean ± SD (range)]	11.6 ± 2.1 (9–17)	12.2 ± 1.2 (10–15)	.07
AOFAS-AHS pain [mean ± SD (range)]	28 ± 4 (20–40)	28 ± 5 (20–30)	.71
AOFAS-AHS function [mean ± SD (range)]	42 ± 3 (35–47)	42 ± 4 (27–47)	.76
AOFAS-AHS alignment [mean ± SD (range)]	1 ± 2 (0–10)	1 ± 2 (0–5)	.29
AOFAS-AHS tot [mean ± SD (range)]	70 ± 7 (55–87)	70 ± 7 (47–82)	.47
**Radiographic data**	**Group A (nonoperative)**	**Group B (operative)**	***p-value***
Calcaneal Pitch (°) [mean ± SD (range)]	13.7 ± 3.9 (9–20)	14.7 ± 3.1(11–21)	.48
Meary’s angle (°) [mean ± SD (range)]	12.6 ± 4.3 (8–20)	13.3 ± 4.7 (9–21)	.82
Heel valgus (°) [mean ± SD (range)]	23.7 ± 8.8 (6.6–46.8)	26.4 ± 7.7 (12.8–38.5)	.37
JSN (mm) [mean ± SD (range)]	2.8 ± 1.0 (0.6–4.8)	2.7 ± 0.9 (1.7–4.8)	.28
Rozansky classification	I: 13	I: 18	.19
	II: 9	II: 4	
	III: 12	III: 7	
	IV: 8	IV: 4	
	V: 5	V: 1	

The mean age at treatment was 11.8 years (9–17): 11.6 (9–17) for the nonoperative group and 12.2 (10–15) for the operative group There were no correlations between AOFA-AHS at baseline and sex, age, bilaterality, radiografic features. In all cases, the area of the coalition involved less than 50% of the subtalar joint and no radiographic OA was observed. The mean hospitalization time was 6 days (range 3–10) in the operative group and 4 days (range 1–9) in the nonoperative group. With the numbers available, the difference was not statistically significant (*p*-value = 0.884).

The mean follow-up averaged 6.6 (3–12) years and was significantly longer in the nonoperative group (7.8 versus 4.7 years; *p* < 0.0005), since the operative procedure was increasingly practiced in the latest years.

At the latest follow-up, the AOFAS-AHS significantly increased in both groups, although the operative group showed more pronunced improvements (see Table 2). Also the FADI score was better in the operative group, after adjustment for follow-up duration and IPTW (estimated mean 81 points in the nonoperative group versus 93 points in the operative group. *p*-value < .0005. See Table 3). Return to regular sport activity was possible after an average period of 10 months (range 5–31) in the operative group and after 7 months (range 1–12) in the nonoperative group. (*p*-value = 0.096). With the numbers available, age, gender, AOFAS-AHS at baseline and radiographic parameters did not affect the final outcome.

**Table 2:** Comparison between baseline and latest follow-up AOFAS-AHS in the nonoperative and operative groups. The results are expressed as estimated means

Clinical outcome	Group A (nonoperative)			Group B (operative)			*p-*value
	baseline	follow-up	MD	baseline	follow-up	MD	
AOFAS-AHS pain	28 (26–29)	30* (28–32)	**2 (− 1–5)**	28 (26–30)	37** (34–39)	**9 (6–12)**	***.002***
AOFAS-AHS function	42 (41–43)	43* (41–45)	**1 (− 1–3)**	42 (40–43)	47** (45–49)	**6 (4–8)**	***.04***
AOFAS-AHS alignment	1 (0–2)	5** (4–6)	**4 (3–6)**	1 (0–2)	10** (9–11)	**9 (7–10)**	***.001***
AOFAS-AHS total	70 (68–73)	78** (74–82)	**8 (3–12)**	71 (68–73)	94** (89–98)	**24 (19–29)**	***< .0005***

The estimated means were adjusted by inverse probability of treatment weights (IPTW) and follow-up duration (covariates were calculated at 6.5 years of follow-up), using the patient as random effect to avoid violation of the principle of independence in bilateral cases. 95% confidence interval of the estimated mean is reported in brackets

MD = Mean Difference between baseline and latest follow-up AOFAS-AHS

The asterisks refer to the statistical difference between baseline and follow-up values within the same group. *: difference is significant at *p* < .05. **: difference is significant at *p* < .0005

The *P*-value in the last column is referred to the statistical difference between the MD of the two groups

**Table 3:** Post-operative clinical and functional outcome measured and FADI

Clinical outcome	Group A (nonoperative)		Group B (operative)		*p-*value
	Crude mean	Estimated mean	Crude mean	Estimated mean	
FADI tot	83 (57–100)	81 (78–84)	92 (64–100)	93 (87–98)	<.0005
FADI pain	87 (50–100)	85 (82–100)	94 (69–100)	97 (92–100)	<.0005
FADI function	85 (59–100)	84 (81–87)	93 (64–100)	94 (90–98)	.03
FADI sport	74 (47–100)	72 (68–77)	87 (63–100)	86 (80–93)	<.0005

Group A: non-operative group. Group B (operative group). The results are expressed as crude and estimated means. The crude means are reported as mean and range. The estimated means were adjusted by inverse probability of treatment weights (IPTW) and follow-up duration (covariates were calculated at 6.5 years of follow-up), using the patient as random effect to avoid violation of the principle of independence in bilateral cases. 95% confidence interval of the estimated mean is reported in brackets. The *P*-value in the last column is referred to the statistical difference between the estimated means of the two groups

In the nonoperative group, no complications (such as iatrogenic fractures, compartment syndrome, pressure sores, thermal injuries, dermatitis, deep vein thrombosis, reflex sympathetic dystrophy) were reported, but 6 patients (7 ft) were unsatisfied with the nonoperative treatment and required surgery 2 to 4 years after treatment. In the operative group, we did not report any complication related to the operation. In 4 patients (5 ft) a percutaneous achille’s tendon lenghtening was performed during the operation, in order to achieve 5° of ankle dorsiflexion.

**Fig. 2:**
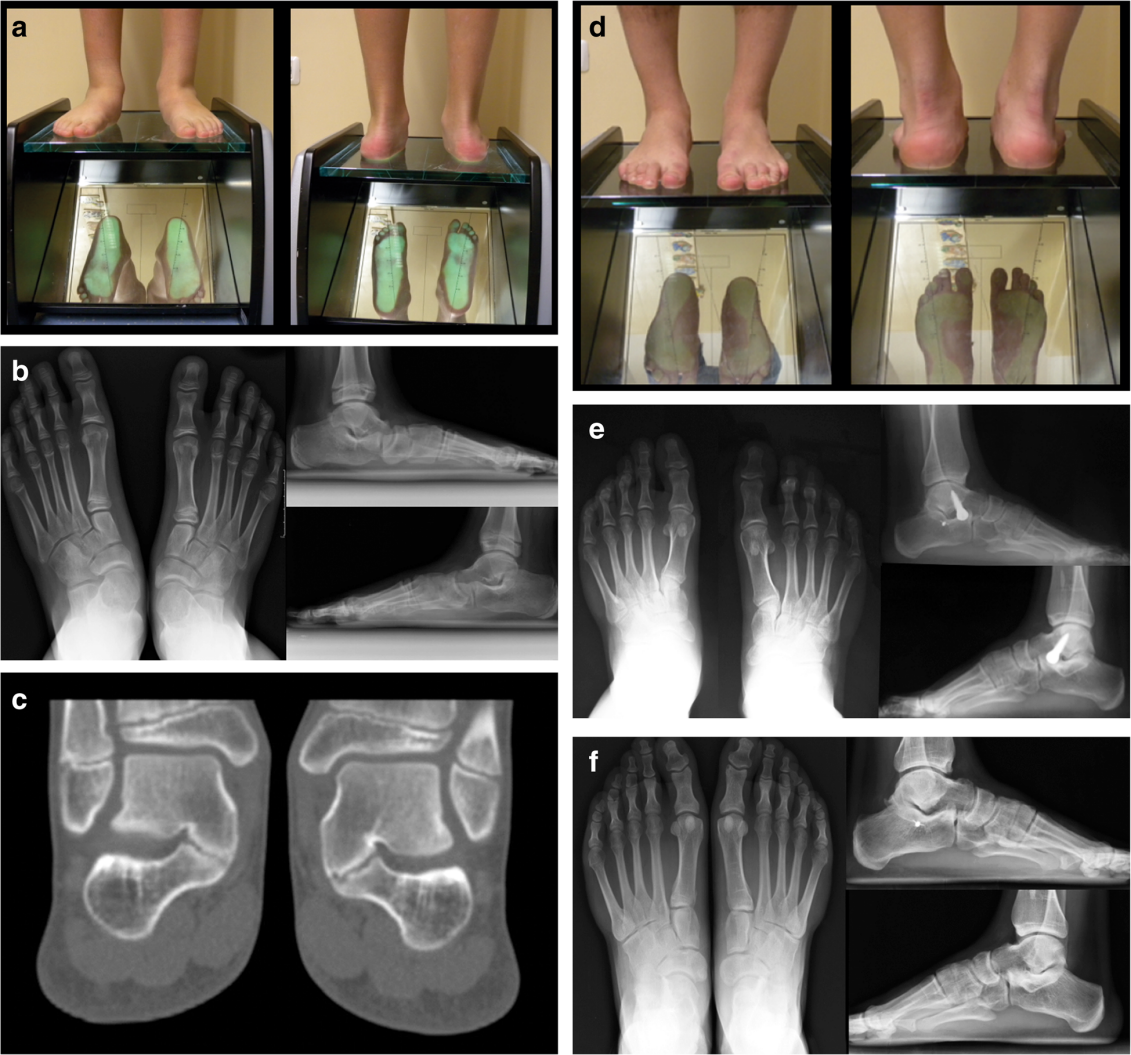
Clinical and radiographic features of 12 years old boy with RFF and TCC. **a** Clinical aspect on podoscope. **b** Antero-posterior and lateral radiographs of the same patient showing the collapse of the longitudinal arch, hindfoot valgus, and forefoot abduction. The “talar beak”, evident on the neck of the talus, suggests the presence of TCC. **c** Coronal CT scan of both feet showing “type I” TCC according to the Rozansky’s classification. **d** Post-operative clinical aspect on podoscope 1 month after surgery. **e** Radiographic aspect showing the screw arthroeresis with correction of the flatfoot. **f** Radiographs 6 years after screw removal, showing that the correction is maintained, and the radiographic parameters are restored

An example is showed in Fig. 2.

## Discussion

To the best of our knowledge, we described the largest case-control study comparing operative and nonoperative treatment of TCC with RFF (see Table 4). We found that operative treatment, consisting in a one-step procedure combining TCC resection, graft interposition and subtalar arthroereisis, may produce better clinical and functional results compared to nonoperative treatment.

**Table 4:** The summarized results of systematic literature review of selected papers. Case reports with less than 3 cases were not reported

Author, Year	N° Patients (N° feet)	Mean age at Treatment	Type of treatment	Follow-up (years)	Rate of good/excellent results	Complications/recurrence
Swiontkowski, 1983 [39]	10 (10)	11–45	Resection (4) Fusion (6)	–	100%	none
Elkus, 1986 [40]	8 ft	13 (8–19)	resection	2 (1–7)	8/8 (100%)	none
Olney, 1987 [33]	9 (10)	14 (10–22)	Resection + fat interposition	3.3	8/10 cases (80%)	1 patient had further surgery for incomplete resection
Scranton, 1987 [41]	14 (23)	24 (11–55)	Cast immobilization (5) Resection (14) Fusion (4)	3.9 (2.2–9.5)	23/23 (100%)	none
Danielsson, 1987 [42]	3 (3)	–	Resection + fat interposition	1.5–14	100%	none
Takakura, 1991 [43]	42 (67)	17.3 (5–54)	a) Nonoperative treatment: 24 (33) b) Operative treatment: 1. resection: 26 (33) 2. fusion: 3 (3).	5.3 (2–11.2)	a) Nonoperative treatment: 68% b) Operative treatment: 83%	a) Nonoperative treatment: residual pain in 8 ft (26%) limited motion in nine feet (29%) b) Operative treatment: mild residual pain in 4/33 ft treated by excision of the coalition (12%) subtalar motion unchanged or decreased in 7/30 ft treated by excision of the coalition (23%) sensory disturbance of the sole in 3/14 ft treated by excision of the coalition (21%) No complications reported in patients treated by subtalar fusion
Salomao, 1992 [44]	22 (32)	14 (10–23)	resection + fat interposition.	2 (1–5.5)	78% of feet became completely painless and 22% achieved relief of pain. Improved deformity in 69% Improved range of motion in 75%.	none
Kumar, 1992 [45]	16 (18)	14 (7–19)	a) resection (3 cases) b) resection + fat interposition (6 cases) c) resection + split flexor hallucis longus tendon interposition (9 cases)	4 (2–8)	12/14 (87.5%)	1 relapse of the coalition with poor clinical outcome
Wilde, 1994 [19]	17 (20)	13 (9–15)	Resection.	1–9	10/20 (50%)	Residual RFF in 10/20 ft (50%)
Kitaoka, 1997 [46]	11 (14)	17 (13–32)	a) resection (9 cases) b) resection + fat or split flexor hallucis longus tendon interposition (5 cases)	6 (2–13)	9/14 (64%)	none
McCormack,1997 [47]	8 (9)	13.6 (10.5–22)	Resection + fat interposition	11.5 (10–16)	7/9 (78%)	none
Comfort, 1998 [48]	16 (20)	14 ± 2	Resection	2.4 (2–6.2)	12/20 (60%)	Four (20%) patients underwent further surgery.
Dutoit, 1998 [49]	8 (9)	14.1	Resection	4.5 (3–11.3)	4/8 (50%)	none
Luhmann, 1998 [50]	20 (25)	12.5 (9–16)	Resection + fat interposition	2.5 (1–8)	19/25 (76%)	2 superfical infection 2 coalition reformation. 5 cases had further surgery (peroneal tendon lengthening, 1 lateral column lengthening 3 arthrodesis)
Raikin, 1999 [51]	10 (14)	12 (9–16)	Resection + split flexor hallucis longus tendon interposition	4.2 (2.7–5)	12/14 (86%)	none
Giannini, 2003 [25]	12 (14)	13 (9–18)	Resection + subtalar arthroereisis by a bioreabsorbable implant	3.3 (3–5.3)	11/14 (79%)	none
Westberry, 2003 [52]	10 (12)	12.7 (9–17.9)	Complete removal of the coalition with removal of the sustentaculum tali	5.1 (1.5–8.7)	9/12 (75%)	One postoperative wound infection. One patient required subsequent lateral column lengthening
Fleming, 2004 [53]	12 (14)	(11–14)	Resection + fat interposition	0.5–2	100%	none
Kernbach, 2008 [26]	3 (6)	14 (12–17)	Resection + flatfoot reconstruction*	3.3 (1.3–4.5)	6/6 (100%)	none
Sperl, 2010 [8]	3 (3)	13.4 (10–15)	Resection + deepithelialized skin flap interposition.	3.3 (0.5–8)	3/3 (100%)	none
Lisella, 2011 [54]	7 (8)	15 (12–18)	Resection + reconstruction	3 (2–5)	8/8 (100%)	1 infection 1 deep vein thrombosis
Mosca, 2012 [13]	8 (13)	13 (10–18)	a) 5 patients (9 ft) with RFF and TCC (coalition area > 50%): CLO + Strayer or TAL** + medial plication. b) 1 patient (2 ft) with RFF and TCC (coalition area > 50%): simultaneous CLO + resection of the middle facet coalition + Strayer. c) 2 patients (2 ft) with residual RFF after the resection of a middle facet tarsal coalition: CLO + TAL + talonavicular arthrodesis (1 ft)	2–15	Group 1: 9/9 (100%) Group 2: 2/2 (100%) Group 3: 1/2 (50%)	Group 1: 1 patient developed pain under the fourth and fifth metatarsal heads on both feet. Grouo 2: None. Group 3: 1 patient underwent talonavicular arthrodesis for symptomatic arthritis
Gantsoudes, 2012 [29]	32 (49)	13	TCC resection + fat graft interposition	3.5	42/49 (84%)	11 ft (22%) underwent a total of 12 secondary procedures involving the lower extremity, including 2 revisions (4%).
Khoshbin, 2013 [22]	11 (13)	12 ± 2.5	resection alone (1) or with interposition of fat/wax graft (7), flexor digitorum Longus (4) or flexor hallucis longus (1)	2.2	13/13 (100%)	none
Jagodzinski, 2013 [55]	8 (9)	15 (11–20)	Arthroscopic resection.	1–5.5	7/9 (78%)	1 patient developed scar sensitivity at one of the portal sites. 1 patient had posterior tibial nerve damage. 1 patient (2 ft) required further surgery (fusion)
De Wouters, 2014 [21]	6 (7)	14 (11–16)	Resection using 3D printed cutting guides + fascia lata allograft interposition.	1.7	7/7 (100%)	none
Kemppainen, 2014 [56]	19 (26)	13.5 (9–17)	Resection with or without intra-operative assessment through a portable CT scanner	2 (0,5–4)	19/26 (73%)	1 case required further surgery
Krief, 2015 [24]	3 (3)	10 (8–12)	Resection + interposition of a sterile silicone sheet	3.3 (1–6.7)	3/3 (100%)	none
Knörr, 2015 [57]	15 (16)	11.8 (8–15)	Arthroscopic resection	2.3 (1–3.7)	16/16 (100%)	Complex regional pain syndrome in 1 patient. No recurrences.
Hamel, 2016 [58]	80 ft	8–17	a) resection + fat interposition (31) b) resection + fat interposition + tarsal osteotomy (26) c) fusion (20) d) fusion + tarsal osteotomy (3)	3	Group 1 27/31 (87%) Group 2 20/26 (77%) Group 3 18/20 (90%) Group 4 3/3 (100%)	3 cases underwent further surgery
Mahan, 2017 [59]	36 (51)	13.1 ± 2.6	resection	2.7	41/51 (80%)	2 patients developed superficial wound infection.
Masquijo, 2017 [60]	13 (14)	14 (11–16)	7 patients (8 ft): simultaneous TCC resection of the coalition and reconstruction; 6 patients (6 ft): isolated reconstruction	3.7.	14/14 (100%)	1: Hardware prominence; 1: superficial infection
Hubert, 2018 [23]	10 (12)	12.2 (10–18)	TCC resection and interposition of pediculated flap of the tibialis posterior tendon sheath	4.8	12/12 (100%)	none
Shirley, 2018 [61]	16 (16)	11.4	Conservative treatment.	1.7 (0.2–7.4)	9/14 (54%)	38% of cases required surgery
Present Study	55 (81)	11.8 (9–17)	Group 1: non operative treatment (47); group 2: coalition resection, graft interposition and subtalar arthroereisis (34)	6.6 (3–12)	26/47 (55%) 26/34 (76%)	No complications, but 6 patients (7) in group 1 were unsatisfied and required surgery

Currently, poor evidence supports the management of painful RFF with TCC in children. Recommended treatment includes manipulation, continuous or intermittent casting and orthosis, while surgery is generally reserved to those cases in which nonoperative treatment fails [1, 13–15, 41].

Concerning the nonoperative treatment, it can relieve pain and improve function in 25–68% of cases [18, 40, 43, 61–63]. Most authors suggested that surgery should be performed on patients whose symptoms were not relieved by conservative treatment. However, previous reports about nonoperative treatment were often weakened by limited statistical analysis or lack of essential outcome measures. We found that nonoperative management produced satisfactory outcomes (total AOFAS-AHS > 80) in 55% of cases and 7 ft (15%) required surgery after nonoperative treatment. These results are consistent with previous studies investigating the role of nonoperative treatment of RFF with TCC [43, 61]. Moreover, in our experience, the manipulation under anesthesia and casting was much more expensive than other nonoperative strategies (for instance, analgesia, physiotherapy and orthotics) since, it contempated an average hospitalization of 4 days and a mean surgical room occupation of 30 min. Therefore we believe that this treatment should be reserved only to those cases in which other nonoperative treatments failed, that need but refuse surgery. We believe that efforts should be done to avoid costly and time consuming nonoperative attempts, if they are destined to fail or to be unsatisfactory for the patient. A possible prognostic factor could be the level of pain at baseline as recently suggested by Birisik [14]; therefore, children with high level of pain could be addressed directly with surgical treatment.

If surgery is considered as definitive management, the surgeon must keep in mind the goals of surgery: to eliminate pain and improve function [13, 64].

Currently, there is no complete agreement concerning the best surgical strategy in children with RFF and TCC. Recommended treatments include bar resection alone or combined with tissue interposition and hindfoot correction [5, 8, 19, 24, 26, 29, 33, 36, 39–57, 59], isolated calcaneal osteotomy [13], subtalar fusion or triple arthrodesis; the latter being recommended for subtalar OA, failure of previous surgeries, or large irresectable coalitions with severe heel valgus [17, 18, 39, 41]. The known poor long-term outcomes of triple arthrodesis, however, make this an undesirable option, particularly for children [16].

Concerning the resection of the coalition, several authors reported favorable results in children with isolated TCC resection.

Wilde et al. reported results from 17 children (20 ft) undergoing TCC resection and fat interposition [19]. He found that heel valgus > 16°, coalition area > 50%, JSN and impingement of the lateral talar process on the calcaneum were predictors of symptoms’ recurrence after surgery. Gantsoudes et al. [29] analyzed a cohort of 32 children (49 ft) treated with TCC resection and fat tissue interposition. They reported satisfactory results in 42 ft (84%), but 11 ft required secondary procedures, in particular 8 corrective osteotomies to realign the hindfoot. The authors aknowledged that a valgus heel could worsen the outcome but they abitually postponed the hindfoot correction, since the use of a cast for eigth weeks could increase the likelyhood of relapse.

Mosca reported outcomes from a cohort of children who underwent isolated calcaneal osteotomy for RFF with TCC, concluding that heel valgus correction may achieve pain relief, whether or not the coalition is resected [13].

Based on our experience, the heel valgus, whenever present, should be addressed along with the TCC, in order to avoid symptomatic recurrence and need for re-operation.

In our practice, the subtalar arthroereisis is the preferred technique to address the heel valgus in children. Currently, this technique is commonly used to address painful flexible flatfoot in children [65–68]. The main advantages include minimal invasiveness, short surgical time, early return to daily activities, favorable and durable results with low rate of complications. The lateral arthroereisis does not burn any bridges for future treatment modalities, making this procedure suitable for children [68]. Compared to the calcaneal osteotomy [13, 29], the screw arthroereisis limits or does not require a long time of cast immobilization [10, 67, 69, 70]. Moreover, there is initial evidence that lateral arthroereisis may offer a potentially less-invasive alternative to lateral column lengthening [71]. On the other hand, potential disadvantages and complications of the subtalar arthroereisis include loosening, breakage of the implant, pain and discomfort at the surgical incision, peroneal spasm, joint effusion, stress fracture and infection [69, 70, 72, 73]. Although there is no evidence about the role of the hardware removal, in our practice we routinely remove the calcaneo-stop screw 2 years after surgery. This procedure maybe reduces the likelihood of breakage or loosening of the screw, residual pain and increase the subtalar motion without significant relapse of the heel valgus deformity.

Some brief reports and short case-series describe the association of TCC resection and hindfoot realignment in children [25, 26, 54, 58, 60].

Giannini et al. investigated 12 children (14 ft) undergoing TCC resection and subtalar arthroereisis by bioresorbable screw, reporting improvement of the subtalar motion in 13/14 patients, complete restoration of alignment in 3 ft, partial in the remaining 11 ft and pain improvement in all cases, at a mean follow-up of 3 years. The authors demonstrated that hindfoot alignment, subtalar motion, and age at surgery were predictors of symptoms’ recurrence after surgery [25]. These findings were confirmed also in other studies, suggesting that, whenever indicated, this kind of surgery should be undertaken at an early age, before the arthritic changes of the subtalar joint might jeopardize the outcomes [19, 27].

Kernback described excellent results in 3 children with RFF and TCC, undergoing combined TCC resection and calcaneal osteotomy [26].

To the best of our knowledge, we presented the largest series of RFF with TCC in children, comparing nonoperative and operative management. Nonetheless, this study has weaknesses. The retrospective design and lack of randomization introduced potential biases. In particular, the follow-up period was different between the two groups and insufficient for the potential onset of subtalar OA, especially in the operative group. We performed propensity analysis and statistical adjustment to correct or mitigate biases, nonetheless the concern remains. Few postoperative radiographs were available, therefore, no conclusion could be drawn about radiographic correction, recurrence of coalition, and onset of radiographic OA.

The AOFAS-AHS is a clinician-based outcome measure, which lacks sufficient reliability, validity and numeric threshold for a clinically significant difference [74].

To overcome this issue, we administered the FADI at the latest visit, but the lack of a preoperative patient-reported measure limits any consideration about the real effectiveness of both treatments from the patient’s perspective. The study compared two possible ways to manage RFF and TCC, thus it cannot completely answer to some important questions such as the role of manipulation over just immobilization, the risk-effectiveness and cost-effectiveness of the anesthesia, the effect of the arthroereisis over just resection and the comparison with other surgical procedures, such as osteotomies. The allograft interposition possibly reduces the rate of relapse and increases subtalar motion but increases the costs of the procedure; therefore, additional studies must be conducted to demonstrate the superiority of the allograft over autograft (fat tissue, tendon sheath), silicone or bone wax.

## Conclusion

Our study describes a one-step procedure combining TCC resection, graft interposition and subtalar arthroereisis. This procedure produced better outcomes in comparison to the nonoperative treatment, increasing subtalar motion and improving foot posture in most cases. Further prospective randomized studies are needed to confirm our findings and to try to identify the best surgical option to treat this condition.

## Data Availability

The datasets used and/or analyzed during the current study are available from the corresponding author on reasonable request.

## Abbreviations

RFF: Rigid flatfoot
FFF: Flexible flatfoot
TCC: Talocalcaneal coalition
CNC: Calcaneonavicular coalition
CT: Computed Tomography
AOFAS-AHS: American Orthopaedic Foot and Ankle Society – Ankle Hindfoot Score
JSN: Joint space narrowing
OA: Osteoarthritis
FADI: Foot and Ankle Disability Index
PS: Propensity Score
IPTW: Inverse probability of treatment weights
